# Relative effectiveness of different resistance training modalities on lower-body strength and explosive power: a systematic review and network meta-analysis

**DOI:** 10.3389/fphys.2026.1823323

**Published:** 2026-06-30

**Authors:** Jinxuan Bao, Xundian Liu, Yaxuan Huang, Xiaoyu Liu, Ziyu Wang

**Affiliations:** 1 School of Strength and Conditioning Training, Beijing Sport University, Beijing, China; 2 Sports Coaching College, Beijing Sport University, Beijing, China; 3 School of Arts and Sciences, Fuyao University of Science and Technology, Fuzhou, China; 4 Faculty of Health, University of Canterbury, Christchurch, New Zealand

**Keywords:** auto-regulated resistance training, autoregulatory progressive resistance exercise, network meta-analysis, percentage-based training, variable resistance training, velocity-based training

## Abstract

**Background:**

Traditional percentage-based training (PBT) prescribes loads according to a fixed percentage of one-repetition maximum (1RM) but does not account for daily fluctuations in performance readiness. In contrast, auto-regulated resistance training (ART)—including velocity-based training (VBT), autoregulatory progressive resistance exercise (APRE), and Rating of Perceived Exertion (RPE)/Repetitions in Reserve (RIR)-based autoregulatory training (RPE-AT)—and variable resistance training (VRT) provide more adaptive load adjustments. However, their relative effectiveness for improving lower-body maximal strength and explosive power remains unclear.

**Objective:**

To systematically evaluate and compare different resistance-training modalities, including APRE, VBT, RPE-AT, VRT, and PBT, on maximal lower-body strength and explosive power in healthy and active individuals using a network meta-analysis (NMA) method, and to provide a ranking of therapeutic effects.

**Method:**

Following the PRISMA-NMA guidelines, a systematic search was conducted across the Web of Science, PubMed, Embase, Scopus, SportDiscus, and ProQuest databases, ensuring a comprehensive and transparent approach to study retrieval. Randomized controlled trials (RCTs) comparing the effects of APRE, VBT, RPE-AT, VRT and PBT on lower extremity performance were included. The quality was evaluated using the Cochrane Risk of Bias assessment tool. Pairwise meta-analysis and NMA under the frequentist framework were conducted using R software. The standardized mean difference (SMD) and its 95% confidence interval (CI) were calculated, and the intervention measures were ranked by the cumulative area under the ranking probability curve (SUCRA).

**Results:**

A total of 27 RCTs involving 694 participants were included, consistent with the sample size and study quantity reported in relevant systematic reviews and meta-analyses of RCTs. For back squat 1-repetition maximum (1RM, a measure of maximal lower-body strength), NMA showed that VRT (SMD = 0.62, 95% CI [0.29, 0.95]), APRE (SMD = 0.59, 95% CI [0.16, 1.02]), and VBT (SMD = 0.41, 95% CI [0.10, 0.73]) significantly improved performance compared to PBT, while RPE-AT (SMD = 0.12, 95% CI [-0.31, 0.56]) showed no significant difference from PBT. The SUCRA ranking for back squat 1RM was: VRT (83.1%) > APRE (79.1%) > VBT (57.1%) > RPE-AT (23.4%) > PBT (7.4%). For countermovement jump (CMJ, a measure of lower-body explosive power), APRE (SMD = 1.08, 95% CI [0.30, 1.87]) and VBT (SMD = 0.62, 95% CI [0.23, 1.02]) were significantly superior to PBT, while VRT (SMD = 0.27, 95% CI [-0.14, 0.69]) showed a non-significant trend toward improvement. The SUCRA ranking for CMJ was: APRE (96.6%) > VBT (74.4%) > VRT (49.7%) > PBT (23.6%) > RPE-AT (5.7%).

**Conclusion:**

APRE, VBT, and VRT were generally associated with more favorable adaptations than PBT in lower-body strength and power outcomes. VRT showed the highest probability of being most effective for back squat 1RM, whereas APRE ranked highest for CMJ performance. These findings provide a comparative synthesis of current evidence and may assist practitioners in selecting resistance training strategies according to specific performance goals, while acknowledging the need for cautious interpretation given variability across studies.

**Systematic Review Registration:**

https://www.crd.york.ac.uk/PROSPERO/view/CRD420251172410, identifier CRD420251172410.

## 1 Introduction

Resistance training is widely acknowledged as a crucial intervention for enhancing maximum muscle strength, explosive power, and overall athletic performance (Kraemer and Ratamess, 2004; Suchomel et al., 2016). Traditionally, Percentage-based training (PBT) has served as the primary framework for developing training programs, wherein the load is determined by a fixed percentage of an individual’s one-repetition maximum (1RM) (Liao et al., 2021). However, this inflexible model does not adequately account for the daily fluctuations in athletes’ physiological states and psychological readiness (McNamara and Stearne, 2010; Saw et al., 2016). Consequently, it may result in insufficient training stimulus during periods of peak condition and excessive training during times of fatigue (Mann et al., 2010), thereby hindering the optimal adaptation of the neuromuscular system and may increase the risk of overtraining.

To address the inherent limitations of Percentage-based training (PBT), a spectrum of more dynamic and responsive training strategies has been developed. Central to this evolution is Auto-regulated resistance training (ART), a paradigm that shifts from predetermined loads to real-time, daily adjustments based on individual performance and status (Larsen et al., 2021; Zhang M. et al., 2022), thereby better accommodating day-to-day fluctuations in neuromuscular readiness. This category encompasses several nuanced methodologies: i) velocity-based training (VBT) utilizes barbell velocity as an objective, biomechanical marker to precisely prescribe and regulate intensity, ensuring the neuromuscular system is stimulated within optimal speed-strength zones (Weakley et al., 2021); ii) autoregulatory progressive resistance exercise (APRE) employs a feedback mechanism where the number of repetitions completed to momentary failure in a dedicated set directly dictates the load for subsequent training sessions, thereby tailoring progression to the individual’s daily capacity (Mann et al., 2010); iii) training guided by the Rating of Perceived Exertion (RPE)/Repetitions in Reserve (RIR)-based autoregulatory training (RPE-AT) leverages subjective fatigue perception to self-regulate intensity, thereby fostering autonomy and aligning effort with recovery status (Greig et al., 2020).

In parallel, variable resistance training (VRT) represents a complementary approach that optimizes the biomechanical stimulus by specifically targeting the inefficiencies inherent to constant external loading. By incorporating elastic bands or chains, VRT dynamically alters the external resistance throughout the range of motion (Shi et al., 2022). This technique is specifically designed to match the human body’s strength curve—where mechanical leverage is poorest in the mid-range (“sticking point”) and strongest at the fully contracted position (Wallace et al., 2006; McMaster et al., 2009). By offloading resistance at the weakest angles and overloading at the strongest, VRT is hypothesized to facilitate a more consistent level of muscular tension across the entire movement (Sawyer et al., 2021). This mechanism not only promotes more complete motor unit recruitment but may also enhance peak force production, thereby establishing VRT as a potent strategy for maximizing strength and power development (Sawyer et al., 2021).

Although both autoregulatory approaches and VRT (McMaster et al., 2009; Larsen et al., 2021) have demonstrated performance benefits, direct head-to-head comparisons among these resistance-training modalities remain limited (Greig et al., 2020). To address this gap with methodological rigor, the present analysis is purposefully scoped. We focus on evaluating two principal lower-body outcomes: the back squat 1RM and the Countermovement Jump (CMJ), as from a practical standpoint, lower-body strength and power are fundamental performance determinants across most sports (Singh et al., 2025), influencing sprinting, jumping, and change-of-direction tasks far more ubiquitously than upper-body measures (Seitz et al., 2014). Moreover, from a methodological perspective the back squat 1RM is a widely used and reliable assessment of multi-joint maximal strength (Grgic et al., 2020), while the CMJ is a sensitive and ecologically valid test for explosive power; their widespread use ensures data homogeneity (Claudino et al., 2017). Concentrating on this well-defined domain allows for a clearer and more conclusive analysis, avoiding the heterogeneity that would arise from including disparate tests (e.g., bench press 1RM).

The rationale for expecting differential effects lies in their distinct proposed physiological mechanisms (Greig et al., 2020). For example, VRT may be particularly advantageous for enhancing maximal force production through the enhancement of mechanical tension, whereas ART methods, such as VBT and APRE, may better facilitate the improvement of explosive force by optimizing the rate of force application and enhancing neural efficiency. Consequently, elucidating the differentiated effects of these training methods on various indicators of sports performance is essential for providing athletes and coaches with evidence-based options for training programs.

Given the limited number of head-to-head randomized trials comparing all modalities, traditional pairwise meta-analyses are inadequate for generating a comprehensive ranking (Salanti et al., 2008). Consequently, this study employs the advanced statistical technique of Network Meta-Analysis (NMA) to integrate both direct and indirect evidence (Lu and Ades, 2004; Salanti, 2012). It systematically evaluates and compares multiple resistance-training modalities, including APRE, VBT, RPE-AT, VRT, and PBT. The study examines the relative effectiveness of these training methods on maximal lower-body strength (back squat 1RM) and explosive power (CMJ) in healthy, active individuals. Additionally, it provides a comprehensive effect ranking for these training modalities that establishes a precise theoretical foundation for sports training practices.

## 2 Method

This study constitutes a systematic review and network meta-analysis, registered on the PROSPERO platform (Registration Number: CRD420251172410), and adhered to the Preferred Reporting Items for Systematic Reviews and Meta-Analyses-Network Meta-Analyses (PRISMA-NMA) checklist (**Supplementary Table 1**).

### 2.1 Data sources

This systematic review and network meta-analysis retrieved data from six databases, including Web of Science, PubMed, Embase, Scopus, SPORTDiscus, and ProQuest (**Supplementary Table 2**). The search period extended from the inception of records in each database to November 1, 2025, with inclusion criteria restricted to English languages exclusively.

### 2.2 Searching strategy

Two authors (J.B. and X.L.) systematically searched the databases from inception to November 1, 2025, using combinations of keywords related to autoregulated and variable resistance training, including APRE, VBT, RPE/RIR-based autoregulation (RPE-AT), and VRT. The full search strategy is provided in **Supplementary Table 3**. In addition to the electronic search, the reference lists of several review articles were meticulously reviewed, and manual searches were performed to guarantee comprehensive coverage (Greig et al., 2020; Weakley et al., 2021). To verify the accuracy of the search, the two researchers cross-checked the selected keywords (J.B. and X.L.). In cases of disagreement regarding keyword selection, a third researcher made the final decision (Z.W.).

### 2.3 Study selection and eligibility criteria

Two researchers employed an identical search strategy across each database and independently reviewed the titles and abstracts for initial screening and identification of all pertinent studies (J.B. and X.L.). Subsequently, the full texts of these studies were independently evaluated against predefined inclusion and exclusion criteria to determine eligibility. Any discrepancies were resolved through discussions with a third expert (Z.W.). After filtering the literature in each database, the references were imported into EndNote X9 software (Clarivate Analytics, Philadelphia, Pennsylvania, USA) for organization. The duplicate references were removed using the deduplication function of EndNote X9 and manually.

This systematic review and network meta-analysis followed the methods of the Cochrane Collaboration and adhered to the PRISMA-NMA guidelines (Hutton et al., 2015). The PICOS methodology (population, intervention, control, outcome, study design) was applied, as follows: (P) Included population: Healthy and physically active individuals with regular sports, athletic, or resistance-training backgrounds, ranging from novice lifters with structured sports experience to competitive athletes (including trained individuals, students, and adolescents); (I) studies in which APRE, VBT, RPE-AT, or VRT constituted the primary resistance-training intervention targeting lower-body performance outcomes. (C) Control measures: PBT, APRE, VBT, RPE-AT, VRT; (O) Outcome measures: back squat 1RM and CMJ height before and after the intervention; (S) Study design: Randomized controlled trials (RCTs). The specific inclusion and exclusion criteria are shown in **Table 1**.

**Table 1:** Inclusion and exclusion criteria.

Project	Inclusion criteria	Exclusion criteria
P	Healthy and physically active individuals with regular sports, athletic, or resistance-training backgrounds, ranging from novice lifters with structured sports experience to competitive athletes (including trained individuals, students, and adolescents)	Experiencing injuries, illnesses, or other clinical symptoms; The elderly population (aged > 60 years)
I	Studies in which APRE, VBT, RPE-AT, or VRT constituted the primary resistance-training intervention targeting lower-body performance outcomes.	Concurrent training or multi-component interventions (e.g., resistance training combined with concurrent aerobic or extensive sport-specific training that confounds the independent effects of the loading modality)
C	PBT, APRE, VBT, RPE-AT, VRT	Other resistance training
O	Back squat 1RM, CMJ height	Inconsistent indicators: power clean 1RM, deadlift 1RM, front squat 1RM, bench press 1RM
S	RCTs	case analysis, relationship study

PBT, Percentage-based training; APRE, Autoregulatory progressive resistance exercise; VBT, Velocity-based training; RPE-AT, RPE/RIR-based autoregulatory training; VRT, Variable resistance training.

After confirming that all eligible literature had been included in the analysis, two researchers (J.B. and X.L.) independently extracted the data into a Microsoft Excel spreadsheet. The extracted data included the article title, year of publication, author names, participant characteristics (age, sample size, exercise modality, and training experience), training protocol details (intervention period, training frequency, and intervention methods), and pre- and post-intervention outcome data (back squat 1RM and CMJ). All data were presented as mean ± standard deviation and were obtained from the text or tables. Any discrepancies between the two researchers were reviewed and resolved by a third researcher (Z.W.). If the full text or relevant data could not be obtained, the corresponding authors were contacted via email. When the original studies provided only graphical data, GetData Graph Digitizer software (version 2.26, S. Fedorov) was used to extract the mean and standard deviation values.

### 2.4 Definitions and classification of core resistance training methods

To enable consistent intervention coding and interpretation across studies, we provide operational definitions of the resistance-training methods included in this review. We classify approaches according to how intensity and/or volume are prescribed and adjusted: fixed percentage-based prescriptions (PBT), autoregulated methods (APRE, VBT, and RPE/RIR-based autoregulatory training), and variable-resistance modalities (VRT). These definitions guide the categorization of interventions in the analyses that follow.

Auto-regulated Resistance Training (ART) is a resistance training approach that dynamically adjusts key variables (intensity, volume) in real time based on an individual’s immediate performance metrics (e.g., movement velocity) or perceptual cues (e.g., RPE/RIR) to match daily physiological states (Greig et al., 2020; Zijing et al., 2025). Its common forms include Autoregulatory Progressive Resistance Exercise (APRE), Velocity-Based Training (VBT), and RPE/RIR-based autoregulatory training, all tailored to optimize individual training stimuli (Burton, 2021; Zijing et al., 2025).

Autoregulatory Progressive Resistance Exercise (APRE) is an autoregulated resistance training method that dynamically adjusts load (intensity and repetitions, not frequency) based on completed repetitions to adapt to an individual’s daily physical condition (Mann et al., 2010; Suchomel et al., 2021; Zhang et al., 2021). It uses 10RM/6RM/3RM protocols: athletes finish the first two sets at a preset load, perform the third to volitional exhaustion, and adjust the fourth set’s intensity by the third set’s repetitions (increased if exceeding target, reduced if not) (Suchomel et al., 2021; Zijing et al., 2025).

Velocity-Based Training (VBT) is an autoregulated resistance training method that prescribes, monitors, and adjusts load based on the stable relationship between movement velocity, %1RM, repetitions, and neuromuscular fatigue (Greig et al., 2020; Banyard et al., 2021). The method utilizes individualized load-velocity profiles to determine target velocities for a training session. During exercise, movement velocity is monitored in real time using devices such as linear position transducers, and the load is adjusted set-by-set to maintain performance within the predetermined velocity range (Orange et al., 2020; Han et al., 2025). A central operational feature of VBT is the application of a predefined velocity loss threshold (commonly between 10% and 25%) to determine set termination (Held et al., 2021; Rodríguez-Rosell et al., 2021).

RPE/RIR-based autoregulatory training (RPE-AT) is an autoregulatory resistance training method wherein the prescription and adjustment of training load are based on the individual’s subjective assessment of effort. This subjective perception has a well-established relationship with objective training variables, including the percentage of one-repetition maximum (%1RM) and movement performance (Helms et al., 2018; Suchomel et al., 2021). The method employs standardized scales, such as the CR10 scale, to quantify the perceived exertion. A specific application within this framework is the Repetitions in Reserve (RIR) approach, which involves estimating the number of repetitions that could be performed prior to reaching volitional failure (Burton, 2021; Gomes et al., 2022; Robinson et al., 2024).

Variable Resistance Training (VRT) is a modality where external load varies with joint range of motion to match muscle force-producing capacity (changing with biomechanical factors like leverage) (Andersen et al., 2022). Unlike Traditional/Constant Resistance Training (TRT/CRT) with constant load (Andersen et al., 2022; Pan et al., 2025), it uses equipment like elastic bands, chains, or cam-based machines (Arazi et al., 2020; Liu et al., 2024). Its core rationale is reducing load in the “sticking region” and increasing it at muscle’s strongest points for optimal full-movement stimulus (Andersen et al., 2022; Pan et al., 2025).

Percentage-based training (PBT) is a traditional resistance training method where external load is prescribed as a fixed percentage of an individual’s 1RM, determined via pre-intervention assessment (Hickmott et al., 2022). Sessions use these preset relative loads, unchanged until the next 1RM test (Orange et al., 2020; Banyard et al., 2021). It standardizes intensity but does not adjust for daily fluctuations in performance capability (Greig et al., 2020). PBT is often used in periodized blocks, with loads systematically varied (e.g., heavier to lighter in a microcycle) while keeping the same percentage-based intensity for all group participants (Orange et al., 2020).

### 2.5 Assessment of risk of bias

The methodological quality of the included randomized controlled trials (RCTs) was evaluated using the revised Cochrane Risk of Bias Assessment Tool (RoB 2.0). This evaluation concentrated on the primary outcomes of this review: the 1RM for back squats and the height of vertical jumps. Two authors (J.B. and X.L.) conducted the assessment independently, and the findings were summarized at the conclusion of the evaluation, following the standard RoB 2.0 protocol, which encompasses five specific aspects:

1. Bias arising from the randomization process.
2. Bias due to deviations from the intended interventions.
3. Bias due to missing outcome data.
4. Bias in measurement of the outcome.
5. Bias in selection of the reported result.

For each domain, a judgment of “Low risk,” “Some concerns,” or “High risk” of bias was assigned based on the responses to the signaling questions from the Cochrane Risk of Bias tool (RoB 2.0). These domain-level assessments were subsequently utilized to derive an overall risk of bias judgment for each study result. Any disagreements between the two authors were resolved through discussion or, when necessary, by consulting a third author to achieve consensus.

### 2.6 Statistical analysis

All statistical analyses of this systematic review and network meta-analysis were conducted using R software (version 4.5.2; R Foundation for Statistical Computing, Vienna, Austria) in the RStudio integrated development environment. The metafor software package was used to perform pairwise meta-analyses, calculate combined effect sizes, and assess heterogeneity (I² statistics) (Viechtbauer, 2010), Netmeta for performing network meta-analyses, generating network graphs, estimating relative effects, and calculating SUCRA values (Balduzzi et al., 2023), and Robvis (Risk of Bias Visualization) for assessing the risk of bias of the included studies. Additionally, RStudio was used for managing and visualizing the data throughout the process.

According to the current operating guidelines (Shim et al., 2019), pairwise meta-analyses and network meta-analyses were conducted based on the frequentist framework. For the network meta-analysis, the standardized mean difference (SMD) with 95% confidence intervals was used as the effect size, calculated as Hedges’ g to correct for small-sample bias. Effects were interpreted as small (0.2 ≤ SMD < 0.5), medium (0.5 ≤ SMD < 0.8), or large (SMD ≥ 0.8) per Cohen’s criteria (Cohen, 1988). To appropriately manage the multi-arm trial (Pan et al., 2025, which compared 20% VRT, 35% VRT, and a shared PBT control) and strictly preclude unit-of-analysis errors, we adhered to Cochrane methodological guidelines (Higgins et al., 2024). We evenly split the sample size of the shared PBT control group to create two independent pairwise comparisons against the VRT arms, thereby reducing the risk of double-counting control participants within the network, consistent with Cochrane recommendations. The statistical analysis then followed a sequential framework, beginning with visualization of the evidence structure via the network plot, followed by quantification of global heterogeneity using the I² statistic and Cochran’s Q test. Subsequently, the transitivity assumption and agreement between direct and indirect evidence were comprehensively evaluated: node-splitting analysis was employed to detect local inconsistency, loop test was conducted to identify loop inconsistency, and a design-by-treatment interaction test was performed to assess global inconsistency (Jackson et al., 2014). Based on these tests, the following pre-specified decision rule was applied: if no important statistical inconsistency was identified (p > 0.05 for key tests), a consistency model would be used for the primary analysis; otherwise, sources of inconsistency would be explored and an inconsistency model or other alternative strategies would be considered. Treatment hierarchies were then established using cumulative ranking probabilities, visualized through the surface under the cumulative ranking curve (SUCRA) (Veroniki et al., 2016). Finally, funnel plots and Egger’s regression tests were generated to assess potential small-study effects and publication bias across key comparisons. In addition, a leave-one-out (LOO) sensitivity analysis was conducted for all pairwise comparisons of each outcome, with the network re-estimated after sequential removal of one study at a time. Results were presented both as detailed LOO influence plots (showing re-estimated SMDs and 95% CIs after each study removal) and as a robustness summary figure. Robustness was quantified as (1) direction consistency (percentage of iterations maintaining the same SMD sign) and (2) significance consistency (percentage maintaining the same statistical significance status).

## 3 Results

### 3.1 Identification and screening of studies

The total number of retrieval records for various categories (APRE, VBT, RPE-AT, VRT) was 13,138. After removing duplicates (n = 7,109), 6,029 records remained for a preliminary screening. At this stage, review articles and records not relevant to the research topic were excluded (n = 5,980).

**Figure 1:**
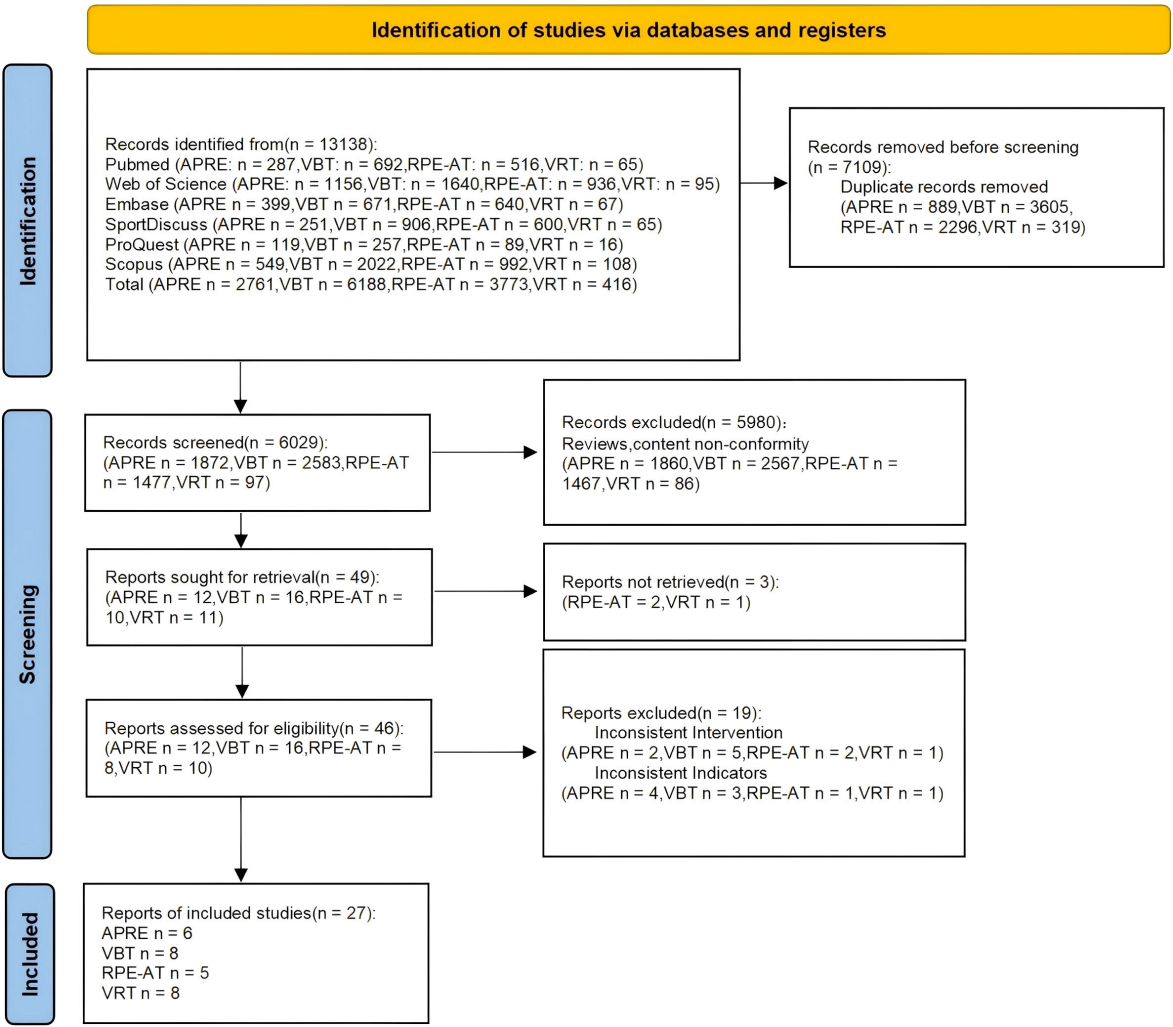
PRISMA flow chart for inclusion and exclusion of studies. The diagram illustrates the number of records identified, screened, assessed for eligibility, and included in the review, along with reasons for exclusion at each stage.

After the initial screening, a total of 49 target articles were determined to be retrieved (12 APRE studies, 16 VBT studies, 10 RPE-AT studies, and 11 VRT studies). Among them, 2 RPE-AT studies and 1 VRT study were not successfully obtained due to resource limitations. A research qualification assessment was conducted on the 46 successfully obtained studies (APRE = 12, VBT = 16, RPE-AT=8, VRT = 10). Studies were excluded based on the criteria of “inconsistent intervention measures” and “inconsistent assessment indicators”. The specific number of excluded studies was: intervention inconsistency (APRE = 2 articles, VBT = 5 articles, RPE-AT=2 articles, VRT = 1 article), indicator inconsistency (APRE = 4 articles, VBT = 3 articles, RPE-AT=1 article, VRT = 1 article). After the above-mentioned full-process screening, 27 valid studies were finally included in this study, including 6 APRE, 8 VBT, 5 RPE-AT and 8 VRT (Mann, 2011; Anderson et al., 2008; Mann et al., 2010; Shoepe et al., 2011; Weber, 2015; Bryce, 2016; Helms et al., 2018; Arazi et al., 2020; Arede et al., 2020; Dorrell et al., 2020; Fernandez Ortega et al., 2020; Orange et al., 2020; Banyard et al., 2021; Graham and Cleather, 2021; Held et al., 2021; Montalvo-Pérez et al., 2021; Sawyer et al., 2021; Gomes et al., 2022; Loturco et al., 2022; Shattock and Tee, 2022; Shi et al., 2022; Zhang M. et al., 2022; Huang et al., 2023; Liu et al., 2024; Rossi et al., 2024; Huang et al., 2025; Pan et al., 2025). The detailed research screening flowchart is shown in **Figure 1**.

### 3.2 Characteristics of the included studies

Following the screening and selection of studies, a total of 27 studies were incorporated into the network meta-analysis (NMA) (Mann, 2011; Anderson et al., 2008; Mann et al., 2010; Shoepe et al., 2011; Weber, 2015; Bryce, 2016; Helms et al., 2018; Arazi et al., 2020; Arede et al., 2020; Dorrell et al., 2020; Fernandez Ortega et al., 2020; Orange et al., 2020; Banyard et al., 2021; Graham and Cleather, 2021; Held et al., 2021; Montalvo-Pérez et al., 2021; Sawyer et al., 2021; Gomes et al., 2022; Loturco et al., 2022; Shattock and Tee, 2022; Shi et al., 2022; Zhang M. et al., 2022; Huang et al., 2023; Liu et al., 2024; Rossi et al., 2024; Huang et al., 2025; Pan et al., 2025), all of which were randomized controlled trials (RCTs). Among these studies, there were 6 focused on APRE, 8 on VBT, 5 on RPE-AT, and 8 on VRT. The complete list of selected studies, along with their corresponding research details, is presented in **Table 2**. Ultimately, the network meta-analysis included 694 participants. The sample sizes of the individual studies ranged from 14 to 57 participants, with ages spanning from 13 to 35 years. Notably, while the majority of cohorts comprised trained or competitive athletes, the network also integrated wide variations in training maturity, encompassing recreationally active college students (novice lifters) (Shoepe et al., 2011) and adolescent athletes (Fernandez Ortega et al., 2020). Additionally, 22 studies provided statistical results regarding training load (Anderson et al., 2008; Shoepe et al., 2011; Bryce, 2016; Helms et al., 2018; Arazi et al., 2020; Dorrell et al., 2020; Fernandez Ortega et al., 2020; Orange et al., 2020; Banyard et al., 2021; Graham and Cleather, 2021; Held et al., 2021; Montalvo-Pérez et al., 2021; Sawyer et al., 2021; Gomes et al., 2022; Loturco et al., 2022; Shattock and Tee, 2022; Shi et al., 2022; Zhang M. et al., 2022; Huang et al., 2023; Liu et al., 2024; Huang et al., 2025; Pan et al., 2025). Intervention durations ranged from 3 to 24 weeks, with most protocols prescribing 2–3 resistance-training sessions per week. In APRE trials, loads were typically set at approximately 10RM or 6RM or 3RM with set-to-failure progressions (Mann, 2011; Mann et al., 2010; Weber, 2015; Bryce, 2016; Huang et al., 2023), whereas VBT interventions mainly relied on velocity-loss thresholds of 10-20% (Dorrell et al., 2020; Orange et al., 2020; Held et al., 2021; Montalvo-Pérez et al., 2021; Zhang M. et al., 2022; Rossi et al., 2024; Huang et al., 2025). RPE-based protocols targeted perceived exertion values between 7 and 9 on the Borg scale (Arede et al., 2020; Graham and Cleather, 2021; Gomes et al., 2022; Shattock and Tee, 2022), and VRT studies implemented elastic bands or chains providing an additional 20-40% of the concentric load (Anderson et al., 2008; Shoepe et al., 2011; Sawyer et al., 2021; Loturco et al., 2022; Shi et al., 2022; Liu et al., 2024; Pan et al., 2025).

**Table 2:** Characteristics of included studies and training interventions.

Study	n	Age (yrs)	Sex	Athletic	Content	Weeks	Freq	Time(min)	RBS (s)	Intensity	Training Load	Outcome
(Huang et al., 2023)	9	21.2 ± 1.39	NR	Badminton	APRE	4	3	NR	NR	50%-100% 6RM	APRE ≈ VBT	CMJ
	9	22.1 ± 1.52			VBT					80% 1RM; VL = 10%		
(Huang et al., 2025)	15	19.8 ± 1.2	14M/16F	Taekwondo	APRE	8	3	NR	NR	10RM,6RM,3RM	APRE < VBT	back squat 1RM, CMJ
	15	19.7 ± 1.5			VBT					70% 1RM(0.48 - 0.72 m/s) 80%1RM(0.40 - 0.60 m/s) 90%1RM(0.32 - 0.48 m/s)		
(Bryce, 2016)	7	21.57 ± 2.87	M	Resistance- trained males	APRE	6	3	60-75	NR	10RM,6RM,3RM	APRE ≈ VBT	back squat 1RM
	9	22.78 ± 2.73			VBT					Adjust the load based on the pre-session peak speed (with a 3% change threshold)		
(Weber, 2015)	9	20.4 ± 1.6	M	Collegiate Wrestlers	APRE	8	NR	NR	NR	10RM,6RM,3RM	Not reported	back squat 1RM
	9	20.0 ± 1.1			PBT					85% 1RM		
(Mann, 2011)	32	19.62	NR	NCAA Division I male football player	APRE	6	1	NR	NR	10RM,6RM,3RM	Not reported	back squat 1RM, CMJ
	25	19.13			PBT					70%–95% 1RM		
(Mann et al., 2010)	12	20.2 ± 1.0	M	division I collegiate football players	APRE	6	1	NR	NR	10RM,6RM,3RM	Not reported	back squat 1RM
	11	20.3 ± 1.6			PBT					70%-85% 1RM		
(Zhang M. et al., 2022)	8	22.0 ± 1.2	F	Basketball (sport-collegiate female players)	VBT	6	2	NR	90-180	65%–95% 1RM; VL = 5%-20%	VBT ≈ PBT	back squat 1RM, CMJ
	7	21.7 ± 2.3			PBT					65%–95% 1RM		
(Orange et al., 2020)	12	NR	NR	Academy Rugby League Players	VBT	7	2	NR	120-180	60%-80% 1RM; VL = 0.06 m/s	VBT ≈ PBT	back squat 1RM, CMJ
	15				PBT					60%-80% 1RM		
(Montalvo-Pérez et al., 2021)	9	22 ± 7	F	Competitive female cyclists	VBT	6	2	NR	120	55%–75% 1RM; VL = 10%	VBT ≈ PBT	back squat 1RM
	8	30 ± 5			PBT					80%–90% 1RM		
(Dorrell et al., 2020)	8	22.8 ± 4.5	M	Resistance-trained males	VBT	6	2	NR	NR	70%–95% 1RM, VL = 20%	VBT < PBT	back squat 1RM
	8				PBT					70%–95% 1RM		
(Fernandez Ortega et al., 2020)	15	13.6 ± 1.2	F	Soccer	VBT	12	3	NR	180	65% 1RM: VL = 20%	VBT < PBT	back squat 1RM, CMJ
	13				PBT					80% 1RM		
(Rossi et al., 2024)	10	22.70 ± 0.82	F	Resistance-trained females	VBT	6	2	NR	120	70%-95% 1RM; VL = 10%	Not reported	back squat 1RM, CMJ
	10	22.69 ± 0.69			PBT					70%-95% 1RM		
(Banyard et al., 2021)	12	25.5 ± 5.0	M	Resistance-trained males	VBT	6	3	NR	120	69.2% 1RM; VL = 0.06 m/s	VBT ≈ PBT	back squat 1RM
	12	26.2 ± 5.1			PBT					59%-85% 1RM		
(Held et al., 2021)	11	19.8 ± 2.3	9M/2F	Highly trained rowers	VBT	8	2	NR	120-180	80% 1RM; VL = 10%	VBT < PBT	back squat 1RM
	10	19.4 ± 1.7	8M/2F		PBT					80% 1RM		
(Shattock and Tee, 2022)	20	22 ± 3	M	Semi-professional rugby union players	RPE-AT	6	3-4	90-120	NR	70-90%1RM;RPE 7-9, RIR 1-3	RPE-AT ≈ VBT	back squat 1RM, CMJ
	20				VBT					70-90%1RM; velocity ranges 0.4-1.25 m/s		
(Arede et al., 2020)	7	15.8 ± 1.3	F	Youth female basketball players	RPE-AT	6	2	NR	120	RPE 7, RIR 3	Not reported	back squat 1RM, CMJ
	7				PBT					80% 1RM		
(Helms et al., 2018)	10	23.8 ± 4.2	M	Resistance-trained males	RPE-AT	8	3	NR	300-420	RIR 5-10	RPE-AT ≈ PBT	back squat 1RM
	11	20.9 ± 1.4			PBT					65%-92.5% 1RM		
(Graham and Cleather, 2021)	15	27.9 ± 5.3	M	Resistance-trained males from various sports	RPE-AT	6	2	NR	120-180	RIR 4,3,2,1,0	RPE-AT ≈ PBT	back squat 1RM
	16	28.3 ± 5.6			PBT					65-95% 1RM		
(Gomes et al., 2022)	10	25.1 ± 3.5	M	Resistance-trained males	RPE-AT	6	2	NR	120	4-15RM;RPE 9	RPE-AT ≈ PBT	back squat 1RM
	10	27.4 ± 3.8			PBT					4-15RM		
(Shi et al., 2022)	11	20.8 ± 1.4	M	Collegiate Basketball Players	VRT	8	2	60	240	80-90%1RM;40%elastic bands	VRT ≈ PBT	back squat 1RM, CMJ
	10				PBT					80%-90% 1RM		
(Pan et al., 2025)	15	21.65 ± 2.20	M	College students	20%VRT	6	2	40	240	80% 1RM;20%elastic bands	VRT ≈ PBT	back squat 1RM, CMJ
	15				35%VRT					80% 1RM;35%elastic bands		
	15				PBT					80% 1RM		
(Loturco et al., 2022)	12	18.5 ± 0.6	M	Soccer players	VRT	4	3	30	NR	0.4-1.2m/s(Weight reduced by 20%);20% elastic bands	PBT > VRT	CMJ
	13				PBT					0.4-1.2m/s		
(Liu et al., 2024)	12	25.74 ± 3.52	F	Boxers	VRT	5	2	100	120	80%-85%1RM;15 - 20% elastic bands	VRT > PBT	back squat 1RM, CMJ
	12	24.87 ± 2.89			PBT					85% 1RM		
(Arazi et al., 2020)	12	23.75 ± 3.64	F	Untrained	VRT	8	3	45-75	120-180	65%-80% 1RM; 15% chains	VRT ≈ PBT	back squat 1RM
	12	23.58 ± 3.84			PBT					65%-80% 1RM		
(Anderson et al., 2008)	18	20 ± 1	NR	Men’s basketball and wrestling; Women’s basketball and hockey	VRT	7	3	NR	120-180	72%-98% 1RM; 20% elastic bands	VRT≈PBT	back squat 1RM
	21		NR		PBT					72%-98% 1RM		
(Shoepe et al., 2011)	10	20.0 ± 1.4	5M/5F	Recreationally active college students (novice lifters)	VRT	24	3	75	NR	67%-95% 1RM; 20%-35% elastic band.	VRT≈PBT	back squat 1RM
	10	19.9 ± 1.2	5M/5F		PBT					67%-95% 1RM		
(Sawyer et al., 2021)	20	18–25	M	Male DIII collegiate football players	VRT	3	3	NR	NR	50-93% 1RM; 20% elastic band	VRT ≈ PBT	back squat 1RM
	20				PBT					50%-93% 1RM		

PBT, Percentage-based training; APRE, Autoregulatory progressive resistance exercise; VBT, Velocity-based training; RPE-AT, RPE/RIR-based autoregulatory training; VRT, Variable resistance training; RBS, Rest between sets; NR, Not Reported; M, Male; F, Female; Freq: Frequency.

### 3.3 Risk of bias in the included articles

**Figure 2:**
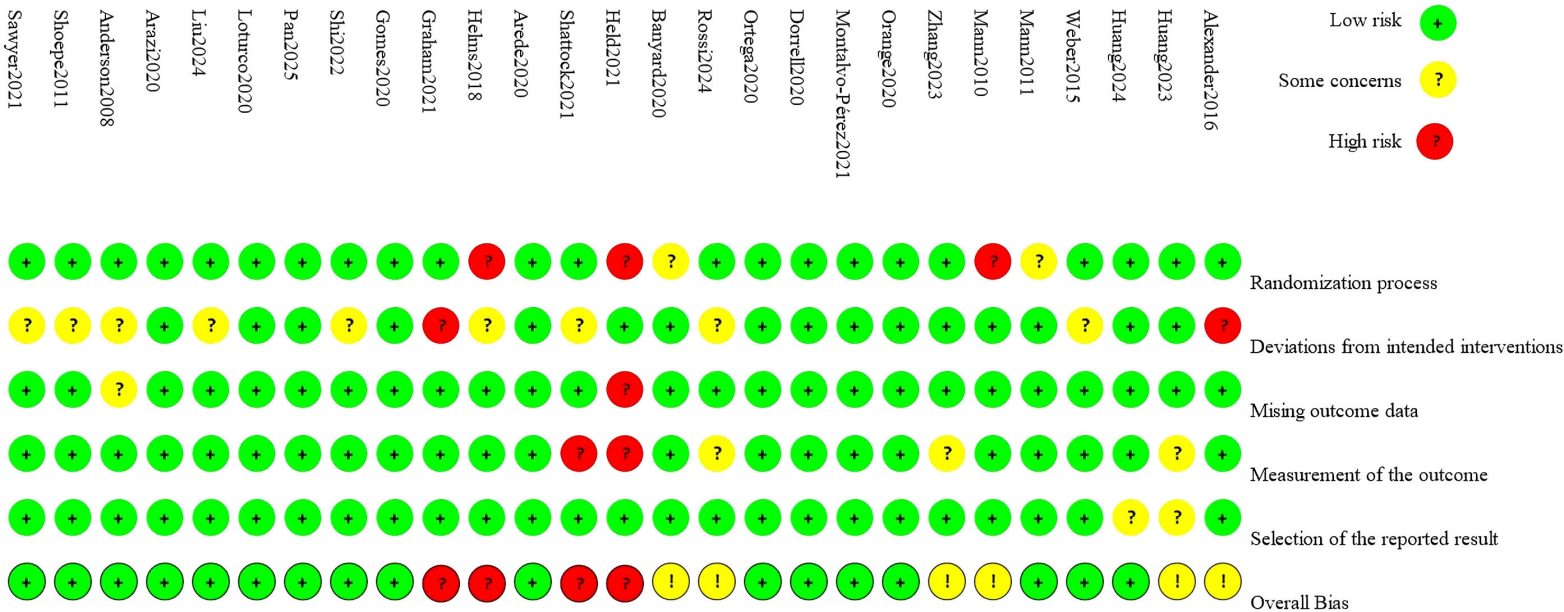
Risk of bias summary assessed using the Cochrane RoB tool for included randomized controlled trials.

**Figure 3:**
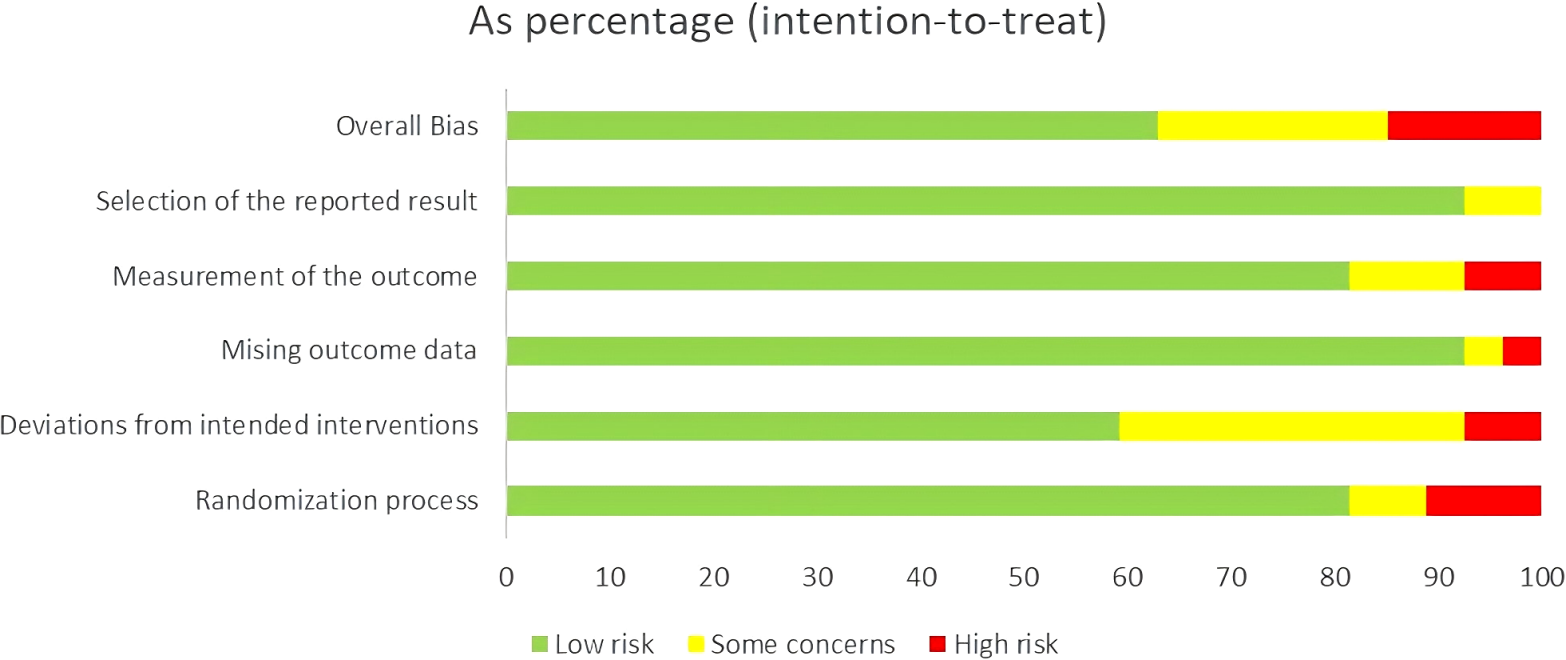
Risk of bias graph for included randomized controlled trials, assessed using the Cochrane RoB tool.

The evaluation results are summarized in **Figure 2**, and the overall distribution of risk-of-bias judgments is presented in **Figure 3**. The findings indicate that the majority of the included studies were rated as high quality. A common issue identified across many studies was the difficulty in maintaining blinding for participants and staff concerning the training intervention measures (Domain 2) (Anderson et al., 2008; Shoepe et al., 2011; Weber, 2015; Bryce, 2016; Helms et al., 2018; Graham and Cleather, 2021; Sawyer et al., 2021; Shattock and Tee, 2022; Shi et al., 2022; Liu et al., 2024; Rossi et al., 2024), which is a frequent limitation in sports training research. Additionally, five studies raised concerns about the randomization process (Domain 1) (Mann, 2011; Mann et al., 2010; Helms et al., 2018; Banyard et al., 2021; Held et al., 2021), often due to insufficiently detailed descriptions of the sequence generation methods or allocation concealment. Overall, four studies were judged to be at high risk of bias, while the remaining studies were classified as having either low risk or some concerns (Helms et al., 2018; Graham and Cleather, 2021; Held et al., 2021; Shattock and Tee, 2022).

### 3.4 Network meta-analysis

#### 3.4.1 Back squat 1RM

**Figure 4:**
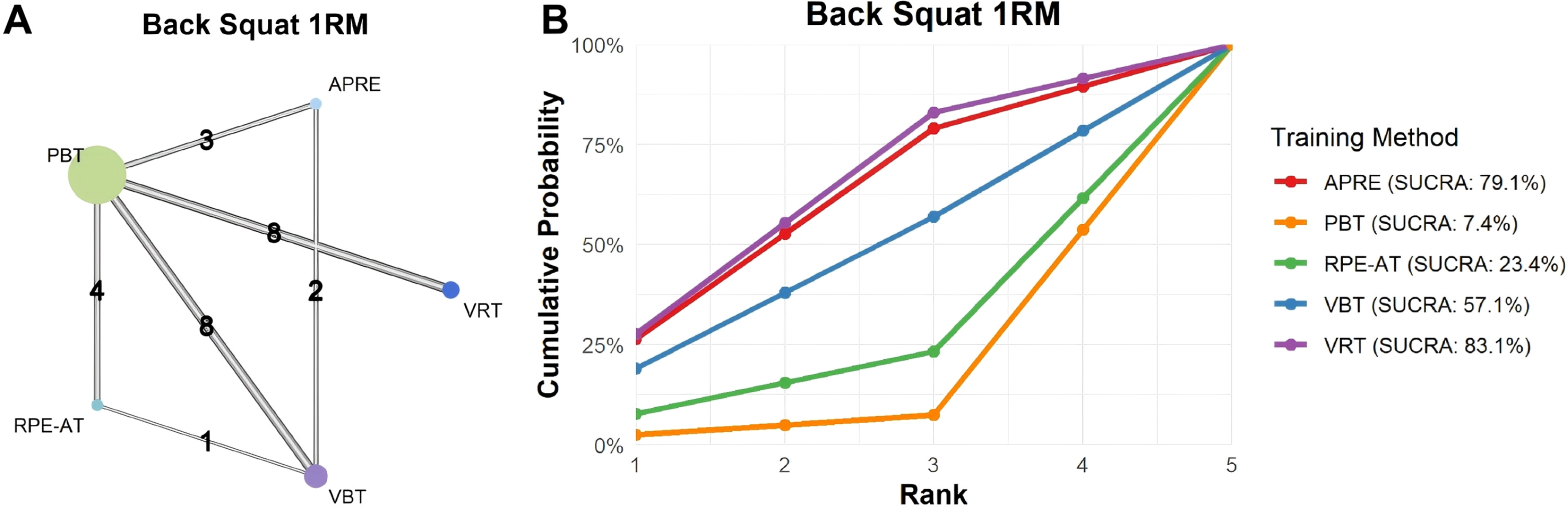
Network analysis results of BS 1RM. Panels **(A, B)** illustrate the network meta-analysis outcomes for BS 1RM performance: **(A)** network diagram depicting direct comparisons between training methods (APRE, PBT, RPE-AT, VBT, and VRT), with edge numbers representing the number of studies comparing each pair; **(B)** cumulative probability plot showing the ranking of training methods based on their effectiveness for improving BS 1RM, with corresponding SUCRA (Surface Under the Cumulative Ranking) values indicating the overall superiority of each method.

**Figure 5:**
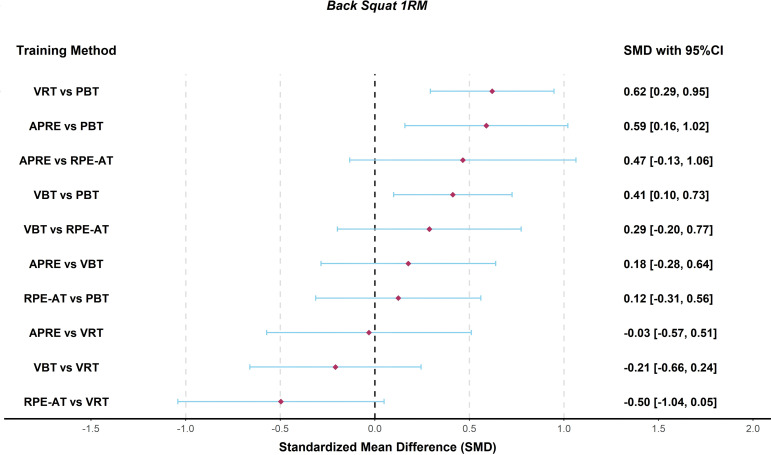
Forest plot of all pairwise comparisons in the network meta-analysis of BS 1RM. The plot presents the standardized mean difference (SMD) with 95% confidence intervals (95% CI) for each pairwise comparison of training methods (VRT, APRE, VBT, RPE-AT, PBT) on BS 1RM performance under a random-effects consistency network meta-analysis (NMA). The vertical dashed line denotes the no-effect threshold (SMD = 0), where positive SMD values indicate a beneficial effect of the first-named training method relative to the comparator, and negative values indicate a beneficial effect of the comparator.

Twenty-five studies contributed eligible back squat 1RM outcomes, yielding 26 pairwise comparisons due to the inclusion of one multi-arm study (**Figure 4A**). Huang et al. (2023) and Loturco et al. (2022) were excluded because they reported countermovement jump outcomes exclusively. Network meta-analysis revealed that compared with PBT, VRT (SMD = 0.62; 95% CI 0.29 to 0.95), APRE (SMD = 0.59; 95% CI 0.16 to 1.02), and VBT (SMD = 0.41; 95% CI 0.10 to 0.73) showed greater improvements in back squat 1RM, whereas RPE-AT showed little-to-no difference vs PBT (SMD = 0.12; 95% CI -0.31 to 0.56). In the remaining pairwise comparisons: Compared to RPE-AT, both VRT (SMD = 0.50; 95% CI -0.05 to 1.04) and APRE (SMD = 0.47; 95% CI -0.13 to 1.06) appeared superior, although the confidence intervals crossed zero. VBT (SMD = 0.29; 95% CI -0.20 to 0.77) also showed a non-significant advantage over RPE-AT. Comparisons among the other three methods (VRT, APRE, VBT) revealed no statistically significant differences: VRT vs VBT (SMD = 0.21; 95% CI -0.24 to 0.66), APRE vs VBT (SMD = 0.18; 95% CI -0.28 to 0.64), and APRE vs VRT (SMD = -0.03; 95% CI -0.57 to 0.51) (**Figure 5**).

Based on SUCRA (**Figure 4B**), VRT (83.1%) is the most likely to be ranked as the best intervention, followed by APRE (79.1%), VBT (57.1%), RPE-AT (23.4%), and PBT (7.4%).

In the network meta-analysis for Back Squat 1RM improvement, low to moderate heterogeneity was observed (Q = 29.70, df = 22, p = 0.126; I² = 25.9%). Global inconsistency, assessed via the design-by-treatment interaction model, was also not statistically significant (Q = 3.79, df = 2, p = 0.150). Comprehensive node splitting evaluation revealed no significant overall local inconsistency across all direct - indirect comparisons (all p > 0.05) (**Supplementary Table 4**). Similarly, local inconsistency was further evaluated via node splitting. All inconsistency factors (IFs) had 95% CIs including zero, suggesting no evidence of significant local inconsistency. To provide a transparent overview of the evidence structure and strength driving these estimates, a comprehensive League Table detailing the origin of evidence (mixed vs. indirect-only) and the number of direct studies for each comparison is presented in **Supplementary Table 6**.

#### 3.4.2 Countermovement jump

**Figure 6:**
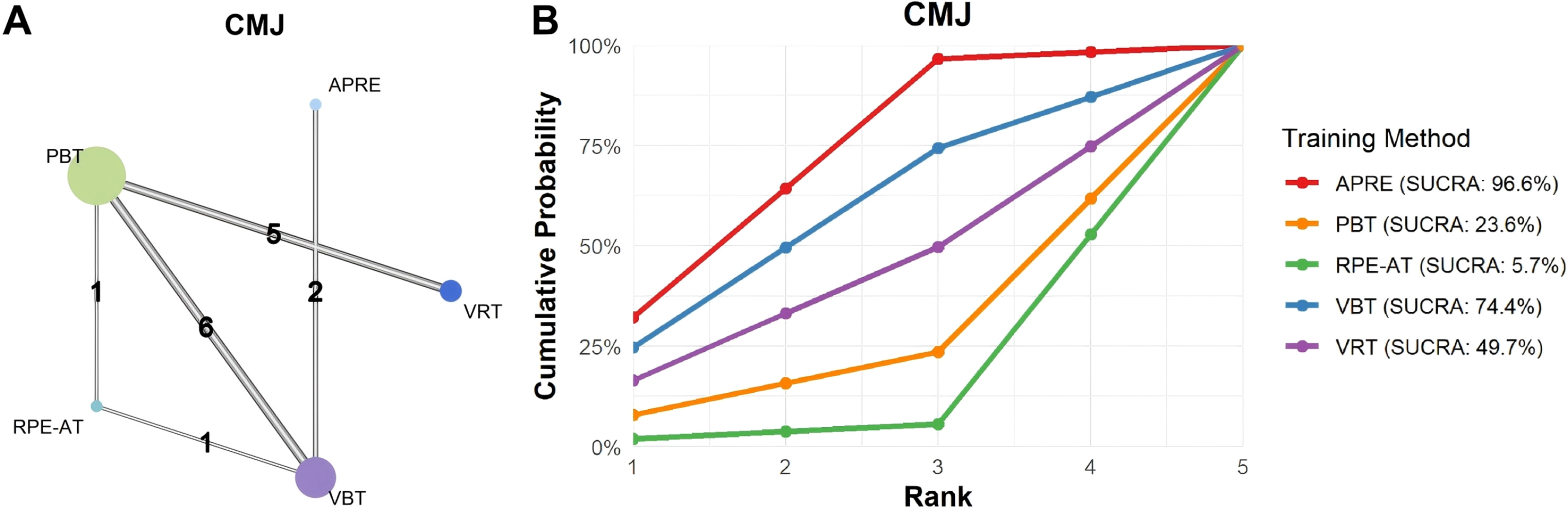
Network analysis results of CMJ. Panels **(A, B)** illustrate the network meta-analysis outcomes for CMJ performance: **(A)** network diagram depicting direct comparisons between training methods (APRE, PBT, RPE-AT, VBT, and VRT), with edge numbers representing the number of studies comparing each pair; **(B)** cumulative probability plot showing the ranking of training methods based on their effectiveness for improving CMJ, with corresponding SUCRA (Surface Under the Cumulative Ranking) values indicating the overall superiority of each method.

**Figure 7:**
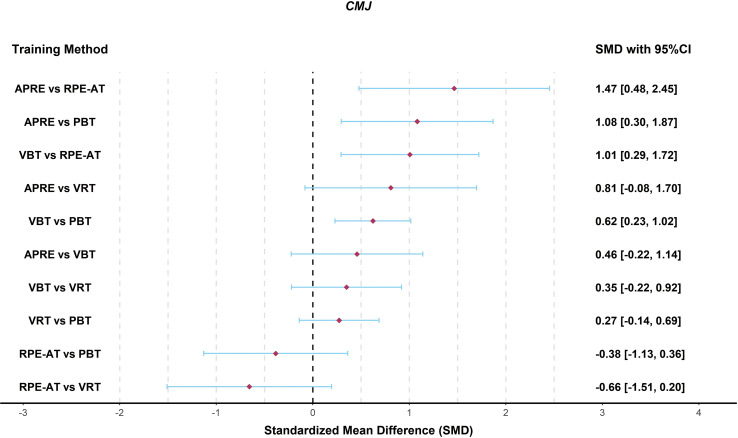
Forest plot of all pairwise comparisons in the network meta-analysis of CMJ. The plot presents the standardized mean difference (SMD) with 95% confidence intervals (95% CI) for each pairwise comparison of training methods (VRT, APRE, VBT, RPE-AT, PBT) on CMJ performance under a random-effects consistency network meta-analysis (NMA). The vertical dashed line denotes the no-effect threshold (SMD = 0), where positive SMD values indicate a beneficial effect of the first-named training method relative to the comparator, and negative values indicate a beneficial effect of the comparator.

We pooled data from 15 studies (**Figure 6A**). Network meta-analysis revealed that compared with PBT, APRE (SMD = 1.08; 95% CI 0.30 to 1.87) and VBT (SMD = 0.62; 95% CI 0.23 to 1.02) showed greater improvements in CMJ, while VRT showed a trend toward greater improvement that was not statistically significant (SMD = 0.27; 95% CI -0.14 to 0.69). In contrast, PBT showed a trend toward being more effective than RPE-AT, although the difference was not statistically significant (SMD = -0.38; 95% CI: -1.13 to 0.36). In subsequent pairwise comparisons: Compared to RPE-AT, APRE (SMD = 1.47; 95% CI 0.48 to 2.45) and VBT (SMD = 1.01; 95% CI 0.29 to 1.72) showed statistically superior effects, whereas VRT showed a positive but non-significant advantage (SMD = 0.66; 95% CI -0.20 to 1.51). Comparisons among APRE, VBT, and VRT revealed no statistically significant differences: APRE vs VBT (SMD = 0.46; 95% CI -0.22 to 1.14), VBT vs VRT (SMD = 0.35; 95% CI -0.22 to 0.92), and APRE vs VRT (SMD = 0.81; 95% CI -0.08 to 1.70) (**Figure 7**).

Based on SUCRA (**Figure 6B**), APRE (96.6%) is most likely to rank the best, followed by VBT (74.4%), VRT (49.7%), PBT (23.6%), and RPE-AT (5.7%).

In the network meta - analysis for CMJ improvement, the Q - test indicated statistically significant heterogeneity (Q = 21.96, df = 11, p = 0.025), and the I² statistic suggested moderate heterogeneity (I² = 49.9%). Global inconsistency, assessed using the design-by-treatment interaction model, was not statistically significant (Q = 6.25, df = 1, p = 0.157). Comprehensive node splitting evaluation revealed that all direct - indirect comparisons showed no significant disagreement (all p > 0.05) (**Supplementary Table 5**). Similarly, local inconsistency was further evaluated via node - splitting. All inconsistency factors (IFs) had 95% CIs including zero, suggesting no evidence of significant local inconsistency. A detailed League Table summarizing the network effect sizes alongside their respective evidence sources (mixed vs. indirect-only) and direct study counts for the CMJ network is provided in **Supplementary Table 7**. The CMJ network comprised 15 studies across 5 nodes. While more compact than the 1RM network, this evidence base is consistent with published network meta-analyses in sports science. Moderate heterogeneity (I² = 49.9%) was appropriately addressed through random-effects modeling. The absence of significant global or local inconsistency supports the validity of the network structure.

### 3.5 Publication bias, meta-regression and sensitivity analyses

**Figure 8:**
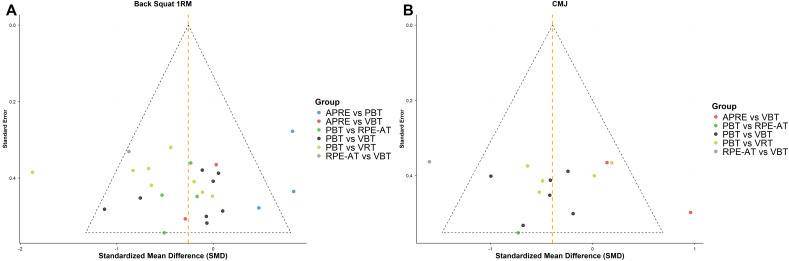
Funnel plots of publication bias assessment from the network meta-analysis (NMA) of Back squat 1RM and CMJ. Panels **(A, B)** display the funnel plots for each outcome under a random-effects consistency NMA: **(A)** Back squat 1RM, plotting standardized mean differences (SMD) against the corresponding standard error of the effect estimates for all pairwise comparisons of training methods; **(B)** countermovement jump (CMJ), with the same SMD versus standard error representation. The dashed lines represent the 95% confidence interval of the expected effect distribution, and the vertical dashed line marks the no-effect value (SMD = 0; positive values indicate benefit). Different colors denote specific pairwise comparison groups.

Funnel plots for Back Squat 1RM and CMJ are presented in **Figure 8**, respectively. Visual inspection of the Back Squat 1RM plot revealed a symmetrical distribution of effect sizes around the pooled estimate. For the CMJ plot, asymmetry was also not observed in the dispersion of effect sizes centered on the pooled estimate. Egger’s regression tests were non-significant for both outcomes (both p > 0.05).

**Figure 9:**
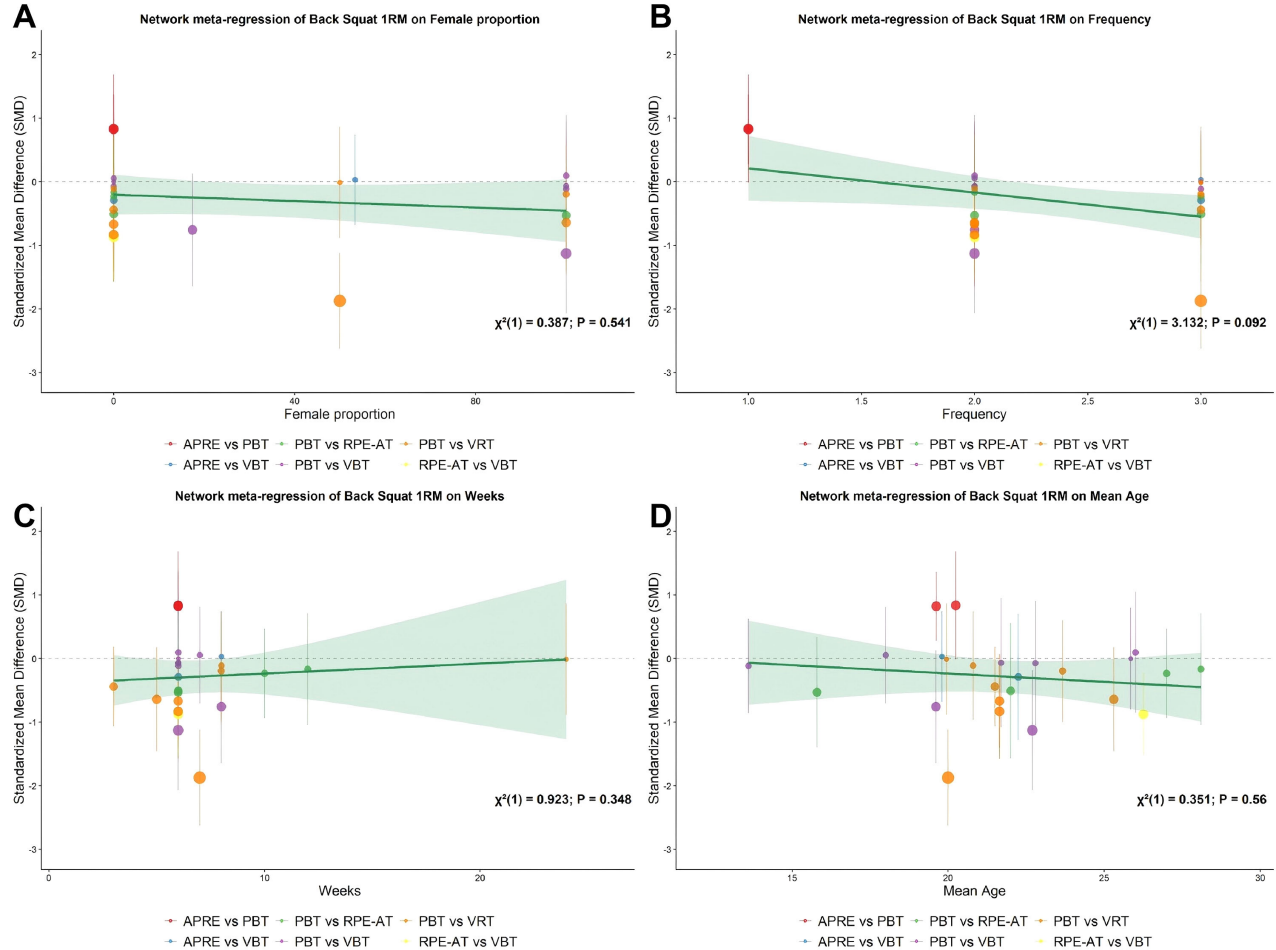
Network meta-regression for Back squat 1RM. Panels **(A–D)** plot the association between study-level covariates and treatment effects under a random-effects consistency NMA with a shared slope across comparisons: **(A)** female proportion (0–1), **(B)** training frequency (sessions/week), **(C)** intervention duration (weeks), and **(D)** baseline mean age (years). The solid line is the fitted common-slope meta-regression; the dashed horizontal line marks the no-effect value (SMD = 0; positive values indicate benefit).

None of the pre-specified study-level covariates (female proportion, mean age, intervention duration in weeks, training frequency in sessions/week) were associated with training effects in the random-effects network meta-analysis (NMA) with a shared slope across comparisons for Back Squat 1RM. The fitted slopes were near zero and the 95% CIs showed no effect (all p > 0.05). Specifically, Joint Wald tests for Back Squat 1RM were: female proportion χ² (1) = 0.387, P = 0.541; frequency χ² (1) = 3.132, P = 0.092; mean age χ² (1) = 0.351, P = 0.56; intervention duration (weeks) χ² (1) = 0.923, P = 0.348 (**Figure 9**).

**Figure 10:**
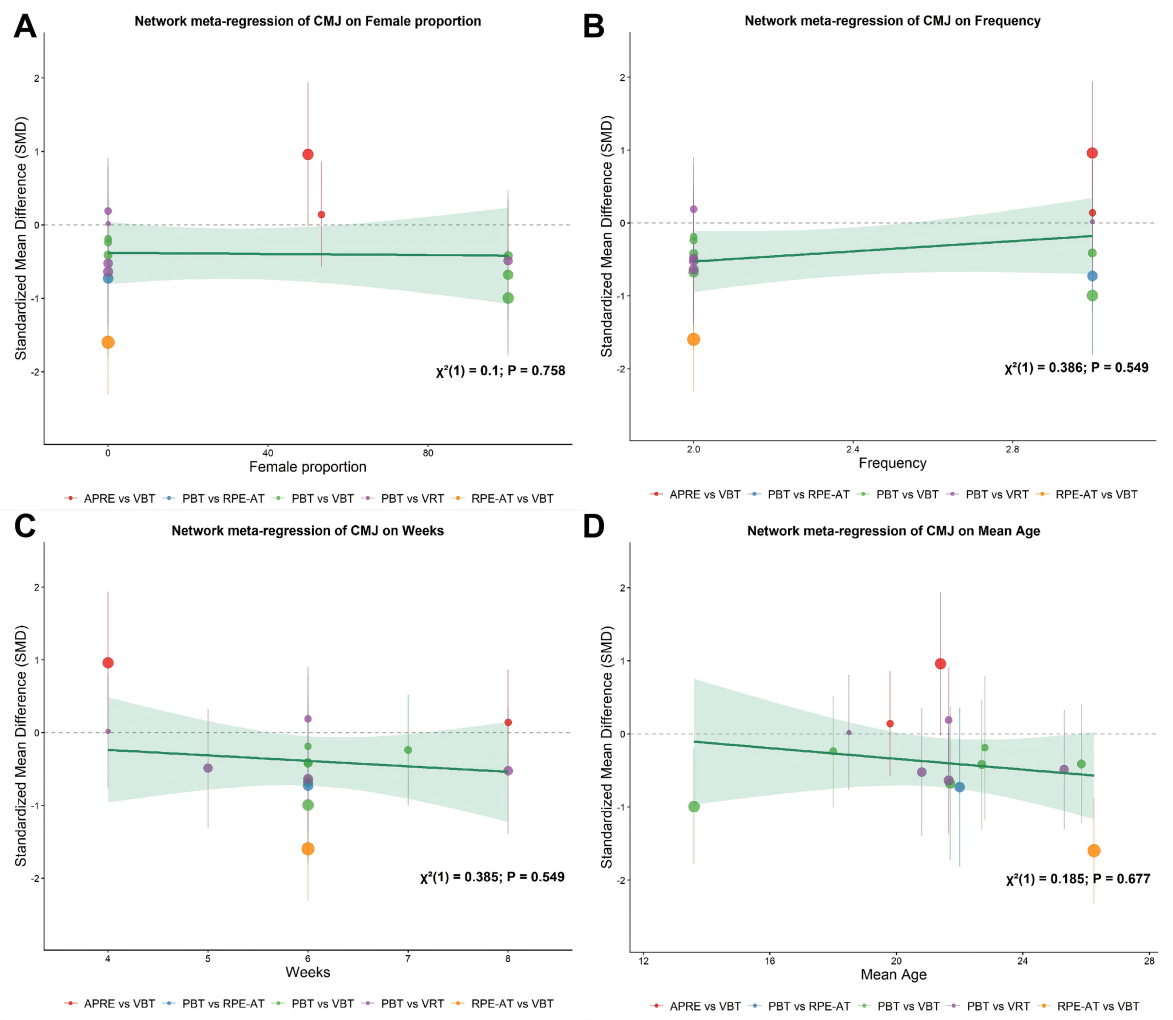
Network meta-regression for CMJ. Panels **(A–D)** plot the association between study-level covariates and treatment effects under a random-effects consistency NMA with a shared slope across comparisons: **(A)** female proportion (0–1), **(B)** training frequency (sessions/week), **(C)** intervention duration (weeks), and **(D)** baseline mean age (years). The solid line is the fitted common-slope meta-regression; the dashed horizontal line marks the no-effect value (SMD = 0; positive values indicate benefit).

Findings were consistent for CMJ: no covariate showed evidence of association with training effects (all p > 0.05). Joint Wald tests for CMJ were: female proportion χ² (1) = 0.1, P = 0.758; frequency χ² (1) = 0.386, P = 0.549; mean age χ² (1) = 0.185, P = 0.677; intervention duration (weeks) χ² (1) = 0.385, P = 0.549 (**Figure 10**). No significant moderating effects were observed for any covariate in either network. These findings indicate that treatment effects are stable across the demographic and protocol characteristics represented in the included studies. However, as with all network meta-regression analyses, the ability to detect small moderating effects is inherently limited by the available study pool (k = 26 for back squat 1RM; k = 15 for CMJ), and the null findings should not be interpreted as definitive evidence of absent covariate effects.

Finally, leave-one-out sensitivity analyses were conducted for all pairwise comparisons of back squat 1RM and CMJ. For 1RM, the consistency of the effect direction ranged from 84.6% to 100%, the consistency of significance ranged from 84.6% to 100%, and marginal significance shifts occurred only for contrasts with baseline 95% CIs near the null. For CMJ, consistency of the effect direction ranged from 93.3%–100%, the consistency of significance ranged from 93.3%–100%, and similar near-null contrast shifts were observed. Heterogeneity showed minimal changes overall, with rare significant reductions in specific deletions. Minor uncertainty widening occurred in a few cases due to reduced network connectivity, but no single study unduly influenced either outcome, confirming robust network stability (**Supplementary Figures 1**-**4**).

## 4 Discussion

This network meta-analysis compared the effects of different resistance-training modalities on lower-body maximal strength (back squat 1RM) and explosive power (CMJ) in healthy active individuals. Overall, APRE, VBT, and VRT were generally associated with more favorable adaptations than traditional percentage-based training (PBT). VRT showed the highest probability of improving back squat 1RM, whereas APRE ranked highest for CMJ performance. These findings provide a comparative overview of current evidence and may help practitioners select training strategies according to specific performance goals.

The superior ranking of VRT for improving back squat 1RM highlights its distinct advantage in maximal strength development. Mechanistically, the incorporation of elastic bands or chains dynamically alters external resistance throughout the range of motion, thereby providing a more consistent mechanical stimulus that aligns with the ascending human strength curve (Sawyer et al., 2021; Shi et al., 2022). This loading profile can enhance muscular tension and maximize motor unit recruitment at the mechanically strongest phases of the lift (Soria-Gila et al., 2015; Andersen et al., 2019). However, the lack of statistically significant differences in the pairwise comparisons among VRT, APRE, and VBT indicates that their relative efficacy should be interpreted with caution rather than as definitive proof of absolute superiority. Conversely, the lower ranking of PBT likely reflects its reliance on rigid, predetermined relative loads that fail to accommodate daily fluctuations in physiological readiness (Greig et al., 2020), potentially leading to suboptimal training stimuli or accumulated fatigue.

Regarding explosive performance, the high rankings of APRE and VBT suggest that autoregulatory approaches are particularly suited for tasks requiring rapid force production and high neuromuscular involvement (Hortobágyi et al., 2021; Suchomel et al., 2021). APRE optimizes training volume and intensity set-by-set based on real-time repetition performance, thereby preserving movement quality and preventing excessive neuromuscular fatigue (Larsen et al., 2021; Huang et al., 2025). Similarly, VBT utilizes real-time barbell velocity as an objective marker to regulate intensity and terminate sets via predefined velocity-loss thresholds, which effectively targets specific velocity-strength zones crucial for jump performance (Banyard et al., 2021; Rossi et al., 2024). In contrast, the less pronounced effect of VRT on CMJ may indicate that its progressive resistance loading profile is less favorable for maximizing movement velocity during purely ballistic, explosive tasks (Loturco et al., 2022). However, it is important to note that the CMJ network comprised fewer studies and exhibited moderate heterogeneity. Given the limited statistical power within this specific network, alongside several non-significant pairwise comparisons among the top-ranked modalities (e.g., APRE vs. VBT), these specific rankings should be interpreted cautiously as probabilistic tendencies rather than definitive conclusions.

A primary strength of this study lies in the application of network meta-analysis to synthesize both direct and indirect evidence across multiple training modalities simultaneously (Christofilos et al., 2022). While traditional pairwise meta-analyses are inherently restricted to isolated, head-to-head comparisons—such as VBT versus PBT (Zhang X. et al., 2022) or VRT versus PBT (Lin et al., 2022)—the current NMA offers a broader, integrated hierarchy of commonly utilized training systems. This comprehensive framework bridges critical evidence gaps where direct trials are scarce, thereby providing a more nuanced decision-making tool for sports science practitioners.

Regarding the validity of the network structure, certain treatment comparisons inherently exhibit structural sparsity or rely on indirect evidence, which increases the uncertainty surrounding specific pooled estimates, as highlighted by the evidence matrices (**Supplementary Table 6** and **7**). Additionally, there is inherent methodological variability within the respective training modalities, such as varying resistance tools in VRT (Liu et al., 2024; Pan et al., 2025) or distinct velocity-loss thresholds in VBT (Dorrell et al., 2020; Held et al., 2021; Huang et al., 2025). While localized diagnostic signals suggested marginal uncertainty in isolated comparisons, the overall structural integrity of the network is strongly supported by comprehensive assessments. Both global and local inconsistency tests revealed no significant friction between direct and indirect evidence, and iterative sensitivity analyses demonstrated that the relative treatment hierarchies remained remarkably stable across these localized variations. Therefore, these findings may be interpreted as relatively consistent probabilistic tendencies rather than being driven solely by isolated statistical fluctuations.

A potential source of clinical heterogeneity within this network stems from the broad inclusion criteria, which spanned a wide spectrum of training maturity—from recreationally active college students (Shoepe et al., 2011) to young adolescent athletes (Fernandez Ortega et al., 2020). From the standpoint of network transitivity, although VRT and autoregulatory modalities utilize fundamentally distinct loading mechanics, they are conceptually unified by their shared objective of optimizing neuromuscular overload, and are robustly anchored by PBT as a common comparator. While this diversity enhances the ecological validity of our findings, it introduces distinct physiological adaptation patterns. Less mature cohorts or novice lifters are highly susceptible to rapid, non-specific early-stage neural adaptations, which may partially dilute the mechanistic differences between advanced modalities. Network meta-regression confirmed that demographic factors and protocol characteristics did not significantly moderate treatment effects, supporting the stability of the network estimates. Nevertheless, because the network exclusively evaluated healthy, physically active individuals (ages 13–35 years), these results cannot be directly extrapolated to clinical, sedentary, or older adult populations.

Additionally, the relatively lower ranking of RPE-AT across both networks warrants objective interpretation. This outcome aligns with previous evidence suggesting that reliance on subjective fatigue perception can introduce greater intra-individual variability than objective autoregulatory metrics, occasionally resulting in suboptimal neuromuscular stimulation compared to precision-driven methods like VBT (Helms et al., 2018; Shattock and Tee, 2022).

In light of these limitations, the present findings should be interpreted as probabilistic comparative evidence rather than definitive declarations of superiority for any single training method. Further large-scale, high-quality randomized controlled trials are warranted to strengthen this evidence base and improve the robustness of future network meta-analyses across diverse demographic profiles.

From a practical standpoint, our findings support a goal-oriented selection of resistance training methods rather than a universally prescribed approach. If the primary objective is to enhance lower-body maximal strength (e.g., for powerlifters or American football linemen), VRT may be preferentially considered, as its mechanical overload is theorized to optimize muscle tension and full-range motor unit recruitment (Sawyer et al., 2021; Shi et al., 2022). Conversely, for improving explosive power and rate of force development (e.g., for basketball players or sprinters), APRE and VBT appear to align more closely with training aims, as their autoregulatory mechanisms help preserve neural efficiency and movement velocity (Banyard et al., 2021; Larsen et al., 2021). Practitioners should note that comparisons among certain top-ranked modalities are partially informed by indirect evidence—a standard feature of network meta-analysis. Therefore, these findings are best applied as evidence-based guidelines (contextualized by the specific evidence structure in **Supplementary Tables 6** and **7**), allowing coaches to flexibly tailor interventions based on an athlete’s specific performance goals and contextual constraints.

## 5 Conclusion

This systematic review and network meta-analysis compared the effects of different resistance-training modalities, including APRE, VBT, RPE-AT, VRT, and traditional percentage-based training (PBT) on lower-body maximal strength and explosive power in healthy, physically active individuals. Overall, APRE, VBT, and VRT were associated with more favorable adaptations than PBT, although their relative effectiveness differed according to the performance outcome assessed. For maximal strength development, measured by back squat 1RM, VRT showed the highest probability of being ranked as the most effective approach, followed by APRE and VBT. These findings suggest that training strategies incorporating biomechanical load modulation or objective autoregulatory feedback may better accommodate individual readiness and neuromuscular demands than fixed percentage-based prescriptions. For explosive power, assessed via countermovement jump performance, APRE and VBT demonstrated a greater likelihood of beneficial effects compared with PBT, while VRT showed smaller and more variable effects. These findings should be interpreted with appropriate consideration of the CMJ network size, though the statistical diagnostics support the validity of the estimates.

Importantly, the observed rankings reflect probabilistic tendencies rather than definitive superiority. Given the limited number of available studies and relatively small sample sizes for several comparisons, these findings should be interpreted cautiously. Taken together, these results support a goal-oriented selection of resistance training methods: VRT may be preferentially considered for maximal strength development, whereas APRE and VBT may be more appropriate for enhancing explosive power. Further high-quality, head-to-head trials are required to confirm these comparative patterns across diverse populations and training contexts.

## Data Availability

The raw data supporting the conclusions of this article will be made available by the authors, without undue reservation.

## Supplementary material

Supplementary Figure 1 Leave-One-Out Sensitivity Analysis of Standardized Mean Differences in Back Squat 1RM Across All 10 Pairwise Training Modality Comparisons.

Supplementary Figure 2 Robustness of Direction and Significance Consistency in Back Squat 1RM Training Modality Comparisons.

Supplementary Figure 3 Leave-One-Out Sensitivity Analysis of Standardized Mean Differences in CMJ Across All 10 Pairwise Training Modality Comparisons.

Supplementary Figure 4 Robustness of Direction and Significance Consistency in CMJ Training Modality Comparisons.

Supplementary Figure 5 Inconsistency Factor Analysis of Back Squat 1RM: Comparing Direct Evidence and Pure Indirect Evidence Across Training Modality Comparisons.

Supplementary Figure 6 Inconsistency Factor Analysis of CMJ: Comparing Direct Evidence and Pure Indirect Evidence Across Training Modality Comparisons.

Supplementary Table 1 PRISMA NMA Checklist of Items to Include When Reporting A Systematic Review Involving a Network Meta-analysis.

Supplementary Table 2 The filters for each database

Supplementary Table 3 Web of science literature selection strategy

Supplementary Table 4 Results of the node-splitting analysis for the back squat 1RM

Supplementary Table 5 Results of the node-splitting analysis for the CMJ.

Supplementary Table 6 Evidence contribution matrix for the back squat 1RM network.

Supplementary Table 7 Evidence contribution matrix for the CMJ network.
